# The Pharmacological NF-κB Inhibitor BAY11-7082 Induces Cell Apoptosis and Inhibits the Migration of Human Uveal Melanoma Cells

**DOI:** 10.3390/ijms131215653

**Published:** 2012-11-23

**Authors:** Shuiqing Hu, Qingqiong Luo, Biyun Cun, Dan Hu, Shengfang Ge, Xianqun Fan, Fuxiang Chen

**Affiliations:** 1 Department of Clinical Laboratories, Ninth People’s Hospital, Shanghai Jiao Tong University School of Medicine, 639 Zhizaoju Road, Shanghai 200011, China; E-Mails: cellhu@gmail.com (S.H.); luoqingqiong2007@126.com (Q.L.); hu_dan_2008@sina.com (D.H.); 2 Department of Ophthalmology, Ninth People’s Hospital, Shanghai Jiao Tong University School of Medicine, 639 Zhizaoju Road, Shanghai 200011, China; E-Mails: catherine1_2_3@163.com (B.C.); geshengfang@hotmail.com (S.G.)

**Keywords:** uveal melanoma, NF-κB, BAY11-7082, apoptosis, cell migration

## Abstract

Uveal melanomas are highly metastatic and have high rate of recurrence due to the lack of effective systemic therapy. The identification of important survival pathways in uveal melanomas provides novel therapeutic targets for effective treatment. In the present study, we found that the NF-κB signaling pathway was constitutively and highly activated in uveal melanoma cells. Treatment with the pharmacological NF-κB specific inhibitor BAY11-7082 markedly decreased the nuclear translocation of NF-κB. In a dose-dependent setting, BAY11-7082 inhibited the proliferation and growth of uveal melanoma cells by inducing apoptosis without effect on cell cycle. The migration capacity of uveal melanoma cells was also significantly suppressed by BAY11-7082 treatment. Mechanistically, BAY11-7082 increased the activity of caspase 3 and reduced the expression of anti-apoptotic protein Bcl-2, but did not influence the expression of pro-apoptotic protein Bax. Furthermore, BAY11-7082 induced uveal melanoma cell apoptosis and inhibited xenograft tumor growth *in vivo*. Collectively, the present study identified NF-κB as an important survival signal for uveal melanoma cells and suggested that administration of specific NF-κB inhibitor BAY11-7082 could serve as an effective treatment for patients with uveal melanoma.

## 1. Introduction

Uveal melanomas, including ciliary body, iris and choroidal melanomas, are the most common primary intraocular tumors in adults [1–4]. Many options are currently available for the treatment of uveal melanomas, such as enucleation, plaque radiotherapy, proton beam radiotherapy and transpupillary thermotherapy. The five-year survival rate for uveal melanoma is 75%, which is comparable to cutaneous melanoma. However, metastases to the liver in uveal melanoma patients remain the leading cause of death. The average survival time for patients diagnosed with liver metastasis is only from 2 to 14 months, and up to 95% of patients with uveal melanoma have already developed liver metastases at the time of death. Therefore, it is an urgent necessity to develop more efficient and novel therapeutic agents for improving the survival of uveal melanoma patients [2,3].

NF-κB, first identified as a nucleoprotein that bound to the enhancer region of the immunoglobulin κ chain gene in B cells, is involved in the regulation of a wide variety of biological responses [5–7]. The mammalian NF-κB family includes five members: NF-κB1 (p50/p105), NF-κB2 (p52/p100), c-Rel, RelA (p65) and RelB. In most cell types, physiologically, NF-κB proteins exist in an inactive state as homo- or heterodimers bound to a family of regulatory IκB proteins in the cytoplasm, including IκBα, IκBβ, IκBγ, IκBε and Bcl-3 [5]. The most common form of NF-κB is the p65/p50 heterodimer, which represents the prototypical complex found in mammals. With the phosphorylation-induced degradation of IκBs by the ubiquitin-proteasome system upon a wide range of stimuli, NF-κB heterodimer is liberated and will translocate from the cytoplasm into the nucleus to act as a transcriptional activator. The active NF-κB could bind to the promoters of a diverse array of genes and regulate the transcription and expression of these genes, including cytokines (e.g., interleukin-1 (IL-1), IL-2, IL-6, TNF-α), chemokines (e.g., MIP-1α, MCP1, RANTES, eotaxin), adhesion molecules (e.g., ICAM-1, VCAM-1, E-selectin), inducible effector enzymes (e.g., iNOS, COX-2) and regulators of apoptosis (antiapoptotic or protective factors) and cell proliferation (e.g., c-IAP1, c-IAP2, XIAP, Bcl-xL, Fas ligand, c-myc, cyclin D1) [5–7].

In the non-disease or resting state, NF-κB response is automatically self-limiting and remains inactive by negative feedback loops. The most common molecule to restrict NF-κB activation is IκBα which masks the nuclear localization sequences in NF-κB proteins [5]. However, NF-κB activity is found to be dysregulated and over-activated in a wide range of solid and hematopoietic malignances, including glioblastoma [8], hepatitis-associated hepatocellular carcinoma [9], colorectal cancer [10], esophageal cancer [11], breast cancer [12], pancreatic cancer [13] and different types of leukemia [14]. A growing body of evidence has suggested that aberrant NF-κB activation correlates with many characteristics and hallmarks of cancer development. Hyperactivation of NF-κB not only contributes to inappropriate local tumor cell survival, growth and proliferation, but also promotes distant metastasis [15].

Given the central role of aberrant NF-κB activation in controlling cancer development and progression, the functional significance of the NF-κB signaling pathway has been intensively investigated and its therapeutic values as a target for cancer therapy have been studied in many types of human cancers. As NF-κB activation is tightly regulated by multi-step signaling pathways, several studies have addressed to target different check points of the signaling process [16–18]. For example, anti-inflammatory and immunomodulatory drugs and natural compounds may inhibit NF-κB by inhibiting IKK activity [4,16]. Another approach of NF-κB inhibition by using proteasome inhibitors is to prevent the proteasome degradation of IκBs, NF-κB1/p105 or NF-κB2/p100 [4,16]. Though there are some translational problems from laboratory bench work to clinical usage, the strategies inhibiting various signaling pathways, particularly NF-κB, have shown some promising effects in the treatment of many different cancers. NF-κB expression and activation was reported in uveal melanoma cells [19]. However, the detailed roles and effects of NF-κB in uveal melanoma cells have not been well investigated and clarified [19].

In the present study, the activation status of the NF-κB signaling pathway in uveal melanoma cells was evaluated and the antitumor effects of a specific NF-κB inhibitor BAY11-7082 were explored and investigated.

## 2. Results

### 2.1. NF-κB Is Constitutively Activated in Uveal Melanoma Cells

**Figure 1:**
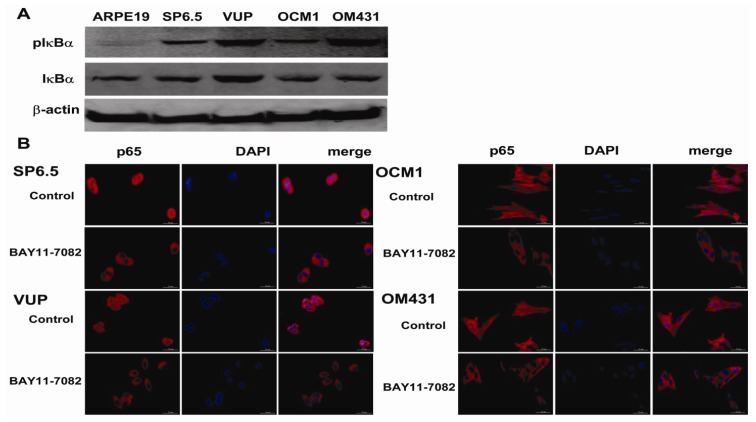
NF-κB activation in human uveal melanoma cells. (**A**) Whole cell lysates were prepared and the expression levels of IκBα and pIκBα were assessed by western blot analysis. β-actin was used as a control to ensure an equal amount of loaded protein. (**B**) Immunofluorescence staining of p65 in uveal melanoma cell lines treated with or without BAY11-7082 as described previously. P65 was labeled red, and nuclei were counterstained with DAPI (blue). P65 was expressed both in the cytoplasm and the nucleus of the resting tumor cells (control), while BAY11-7082 inhibited the translocation of p65 into the nucleus (200× magnification). Data is representative of three independent experiments.

NF-κB is a ubiquitously expressed transcription factor that is regulated by the cytoplasmic inhibitor protein IκBα, which inhibits NF-κB translocation by masking the nuclear localization signals of NF-κB proteins and keeping them sequestered in an inactive state in the cytoplasm [5]. Basal pIκBα was observed significantly in the four untreated uveal melanoma cell lines, SP 6.5, VUP, OCM1 and OM431, while the retinal pigment epithelium (RPE) cell line ARPE-19 showed weakly activation, which meant the constitutive expression and activation of NF-κB in the uveal melanoma cells (Figure 1A). To further confirm the constitutive activation of NF-κB in uveal melanoma cells, the expression and location of p65, one of the most common subunits of NF-κB, was detected by immunofluorescence. P65 was shown to be present in both cytoplasm and nucleus of the control uveal melanoma cells (Figure 1B). However, with the treatment of NF-κB specific inhibitor BAY11-7082 for 2 h, translocation of p65 in the nucleus decreased significantly as shown by the reduced staining in the nucleus, and relocated in the cytoplasm (Figure 1B). These findings indicated that NF-κB is constitutively activated in the four untreated uveal melanoma cells and the specific inhibitor BAY11-7082 could effectively block its activation.

### 2.2. Potent Uveal Melanoma Cell Growth Inhibition by the NF-κB Specific Inhibitor BAY11-7082

**Figure 2:**
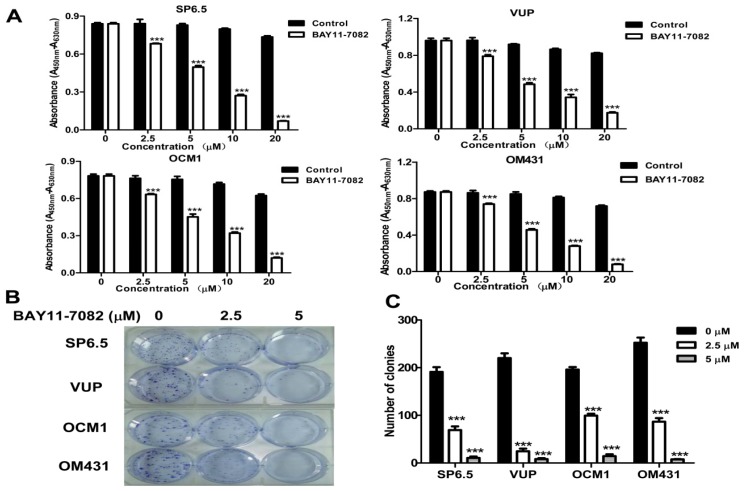
NF-κB blockade by BAY11-7082 inhibited uveal melanoma cell growth and colony formation. (**A**) Cells were treated with different doses of BAY11-7082 as indicated. CCK-8 assay was used to determine the effects of BAY11-7082 on uveal melanoma viability. (**B**) Uveal melanoma cells were seeded into 6-well plates at a density of 300 cells/well and treated with 2.5 μM or 5 μM BAY11-7082 in a 37 °C humidified atmosphere containing 95% air and 5% CO_2_ for three weeks. The cell clones were stained and counted under a microscope at a magnification of 200×. Results are representative of three independent experiments. (**C**) Histograms demonstrated the numbers of colonies in both groups. (***p < 0.001).

The four uveal melanoma cell lines (SP 6.5, VUP, OCM1 and OM431) were treated with varying concentrations of BAY11-7082 for 24 h to test its effects on cell growth that were determined by CCK-8 assay as described previously [20]. As shown in Figure 2A, BAY11-7082 exhibited strong anti-proliferative effects in all four uveal melanoma cell lines in a dose-dependent manner, with approximately 50% inhibition of cell proliferation at the concentration of 5 μM.

Furthermore, BAY11-7082 treatment also significantly inhibited the colony formation capacity of the uveal melanoma cells at the concentration of 2.5 μM (Figure 2B). The number of colonies was reduced in a dose-dependent manner and fewer colonies were formed following treatment with 5 μM BAY11-7082 (Figure 2B,C). These data suggest that NF-κB is constitutively activated in uveal melanoma cells and selective blocking its activation potently inhibits cell growth.

### 2.3. BAY11-7082 Induced Apoptosis in Uveal Melanoma Cells

**Figure 3:**
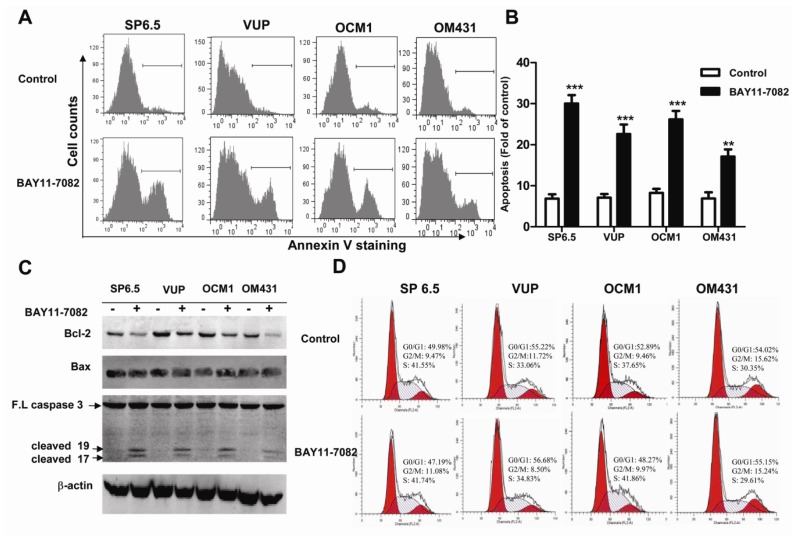
BAY11-7082 induced apoptosis in uveal melanoma cells. (**A**) Uveal melanoma cells were cultured without or with 5 μM BAY11-7082 for 24 h. Apoptosis of uveal melanoma cells was measured by annexin V-staining. (**B**) Histograms demonstrating the percentages of annexin V-positive apoptotic cells. (**C**) Western blot analysis of protein expression of caspase 3, Bcl-2 and Bax in uveal melanoma cells treated with or without BAY11-7082 (5 μM). β-actin was included as a control to ensure an equal amount of loaded protein. Data is representative of three independent experiments. (**p < 0.01, ***p < 0.001) (**D**) Representative cell cycle figures of uveal melanoma cells, which were treated with BAY11-7082 (5 μM) for 24 h.

NF-κB signaling pathway has been shown to regulate cell apoptosis. We therefore investigated whether BAY11-7082 treatment could induce apoptosis of uveal melanoma cells. A significant increase in Annexin V positive cells was determined by flow cytometry when cells were treated with BAY11-7082 (Figure 3A,B). The cleavage form of caspase 3, a specific marker for apoptosis, was greatly increased by BAY11-7082 treatment (Figure 3C), confirming that NF-κB blockade induced uveal melanoma cell apoptosis. NF-κB signaling pathway has been shown to involve in cell apoptosis by regulating the expression of a number of pro-apoptotic and anti-apoptotic genes. Corresponding to the constitutive activation of NF-κB, untreated uveal melanoma cells expressed moderate basal level of anti-apoptotic protein Bcl-2, while BAY11-7082 treatment remarkably reduced its expression (Figure 3C). However, the constitutively highly expressed Bax, one of the pro-apoptotic genes, was not influenced by BAY11-7082 treatment (Figure 3C). Thus, Bcl-2 was decreased by BAY11-7082 treatment and could help to explain why NF-κB blockade induced apoptosis in uveal melanoma cells. The change of cell cycle has also been implicated in regulating uveal melanoma cells proliferation [21–22]. We therefore further studied the effects of BAY11-7082 treatment on cell cycle distribution by flow cytometry. However, there was no significant change in the percentages in each cell cycle phase (Figure 3D). Thus, these data together suggested that BAY11-7082 inhibited the proliferation of uveal melanoma cells by inducing cell apoptosis, but not cell cycle arrest.

### 2.4. Effects of NF-κB Inhibition on Uveal Melanoma Cell Migration

**Figure 4:**
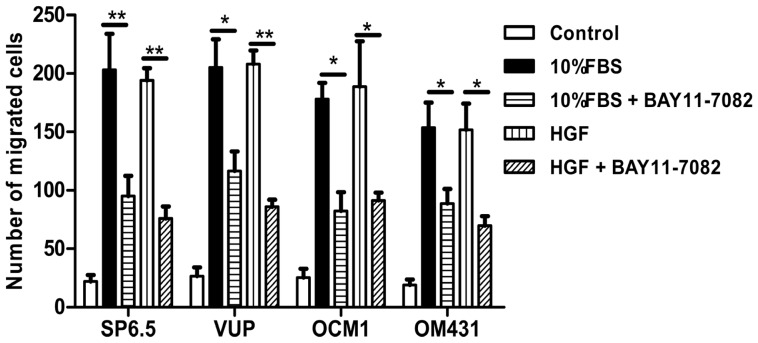
NF-κB blockade by BAY11-7082 reduced the migration of uveal melanoma cells. A transwell migration assay of uveal melanoma cells was performed. The four uveal melanoma cell lines SP6.5, VUP, OCM1 and OM431 were placed in the upper chamber of culture inserts treated with or without BAY11-7082 (5 μM) in DMEM containing 1% FBS and the lower chamber contained DMEM plus 20% FBS or 20 ng/mL HGF in DMEM containing 1% FBS. The number of cells that migrated through pores was quantified by counting five independent visual fields at a magnification of 25× and was expressed as the mean + SD from triplicate experiments. (*p < 0.05, **p < 0.01).

Liver metastases remain the leading cause of death in uveal melanoma patients [3–4]. Identifying signaling pathways that regulate uveal melanoma cell migration would provide therapeutic targets for blocking metastasis. Therefore, we explored whether NF-κB signaling pathway could regulate uveal melanoma cell migration. All the four uveal melanoma cell lines showed very low spontaneous motility, but could significantly migrate to the lower chamber in the presence of high concentrations of FBS or hepatocyte growth factor (HGF), a mesenchymal-derived or stromal-derived multifunctional growth factor which has been shown to promote the migration of uveal melanoma cell lines. SP6.5, VUP and OCM1 cells showed the most potent migration ability, while OM431 cells exhibited moderate migration ability. Treatment of BAY11-7082 at 5 μM significantly suppressed the migration of all four uveal melanoma cell lines to either high concentration FBS or HGF (Figure 4), suggesting that NF-κB pathway was also involved in the regulation of migration and may functionally contribute to liver metastasis of uveal melanoma patients.

### 2.5. BAY11-7082 Suppressed the Growth and Induced Apoptosis in Uveal Melanoma Cells *in Vivo*

**Figure 5:**
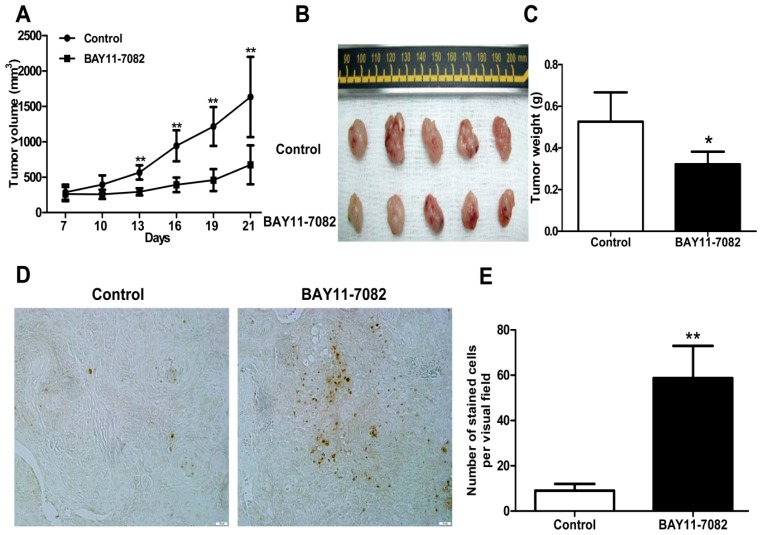
Effects of BAY11-7082 on OCM1 xenograft tumor growth in nude mice. The OCM1 xenograft nude mouse model was established as described in Materials and Methods. Tumor volumes were monitored before treatment. At the end of the experiments, tumor-bearing mice were sacrificed and the tumors were weighed after being separated from the surrounding muscles and dermis. (**A**) The graph represents tumor volumes in mice from the control and BAY11-7082 treated groups. (**B**) Representative images of tumors derived from mice in both groups. (**C**) Tumor weight of mice from the control and BAY11-7082 treated groups. (**D**) Representative images of TUNEL stained apoptotic cells derived from mice in both groups (200× magnification). **E**. Histograms demonstrated the numbers of apoptotic cells per visual field in tumor sections from both groups. (*p < 0.05, **p < 0.01).

To study the therapeutic potential of the NF-κB inhibitor BAY11-7082 in the treatment of uveal melanoma *in vivo*, a xenograft nude mouse model with subcutaneous inoculation of OCM1 cells was established [23–24]. After solid tumors were established and palpable, mice were randomly grouped and 1 μM BAY11-7082 was syringed subcutaneously peri-tumor every day for 14 days. Control mice received the same volume of DMSO. BAY11-7082 treatment significantly suppressed OCM1 tumor growth and tumor volume *in vivo* (Figure 5A,B). Reduced tumor weight was also observed in the BAY11-7082 treated group compared with that of control mice (Figure 5C). To further examine the *in vivo* effects of BAY11-7082 on tumor cell apoptosis, tumor sections from both groups were prepared and examined by TUNEL staining. Remarkably, there were more apoptotic tumor cells in BAY11-7082-treated mice group, as indicated by the increased brown nuclear staining (Figure 5D,E). Therefore, blockade of NF-κB signaling pathway not only suppressed uveal melanoma tumor cell growth and survival *in vitro*, but also induced apoptosis and inhibited tumor growth *in vivo*.

## 3. Discussion

The NF-κB signaling pathway has been proved to be crucial in cancer development and progression, including proliferation, survival, angiogenesis and metastasis [5–7]. Although NF-κB transcription factor family genes including p65, NF-κB1, RelB, NF-κB2 and NIK were found to be expressed in both primary and metastatic uveal melanoma [19], the intact function, detailed activation and underlying mechanisms in uveal melanoma have not been well illustrated. In the present study, we found constitutive expression of pIκBα and p65 localization in both the cytoplasm and nucleus of the four untreated uveal melanoma cell lines, but not in the retinal pigment epithelium cell line ARPE-19. These findings not only confirmed the expression of NF-κB in uveal melanoma cells, but also indicated that NF-κB was constitutively activated in uveal melanoma cells. We further provided for the first time that NF-κB specific inhibitor BAY11-7082, an irreversible inhibitor for IκBα phosphorylation and subsequent proteasome degradation [25], could block the translocation of NF-κB p65 subunit into the nucleus of uveal melanoma cells and markedly inhibit tumor growth by directly inducing uveal melanoma cells apoptosis both *in vitro* and *in vivo*. BAY11-7082 also inhibited the migration of uveal melanoma cells towards high concentrations of FBS and the uveal melanoma cell specific chemoattractant HGF. In a xenograft nude mouse model, we proved that BAY11-7082 effectively inhibited tumor growth by inducing apoptosis of uveal melanoma cells *in vivo*.

Apoptosis is a vital and tightly controlled process and regulated by a balance between both pro-apoptotic and anti-apoptotic genes. Many cancer cells are frequently resistant to apoptosis as a consequence of increased expression of anti-apoptotic proteins or decreased activity of pro-apoptotic proteins. The Bcl-2 protein family is one of most well-established cardinal regulators of cell viability and plays a central role in the control of the apoptotic responses. The ratio of anti-apoptotic (such as Bcl-2) to pro-apoptotic (such as Bax) members may be considered to determine the susceptibility to cell death. Several groups have convincingly demonstrated that blocking NF-kB activity could increase cell death either alone or in response to some stimuli. NF-κB inhibition is associated with decreased Bcl-2 expression under some circumstances [26–27]. Corresponding to the basal and constitutive NF-κB activity, we found that untreated uveal melanoma cells constitutively expressed a moderate level of Bcl-2, while treatment with BAY11-7082 reduced its expression. Preclinical studies suggested potential benefit of Bcl-2 inhibitors for targeted therapy. In fact, Bcl-2 has been shown highly expressed in virtually all cutaneous melanomas and Bcl-2 overexpression has been reported to be associated with an unfavorable outcome in cutaneous melanomas [4]. However, Bcl-2 expression level was not well correlated with clinical or histologic prognostic parameters or survival in uveal melanoma [4]. Nonetheless, the inhibition of anti-apoptotic Bcl-2 by BAY11-7082 may contribute to its effects to induce apoptosis of uveal melanoma cells. The expression of Bax, one of the pro-apoptotic genes, was not influenced by BAY11-7082 treatment. In fact, NF-κB may influence the expression of Bax in a cell type-specific manner. It has been shown that the inhibition of NF-κB activation can lead to increased Bax expression in some cancer cell lines, such as HCT116, OVCAR-3 and MCF7 cells, but not in HCT15 and MCF7 A/Z cancer cell lines [26]. Actually, several transcription factors could bind and regulate the *bax* gene promoter and NF-κB presumably influences only some of them. Altogether Bax expression was not altered, the down-regulation of Bcl-2 expression and the decreased ratio of Bcl-2/Bax might be mechanistically responsible for the reduced uveal melanoma cell survival following BAY11-7082 treatment.

Targeting the cell cycle is an attractive approaches in cancer treatment [28]. Many anticancer agents such as chemotherapeutic drugs have been found to induce cell cycle arrest. Although other studies found that the uveal melanoma cell cycle was regulated by different drugs, microRNAs, and the inhibition of some signaling pathways [21,29], NF-κB blockade by BAY11-7082 did not change the cell cycle profile of uveal melanoma cells. In fact, BAY11-7082 treatment also did not change the expression of cyclin D1 (data not shown), one of the key regulator proteins and whose activity is required for cell cycle G1/S transition.

Approximately more than half patients with primary uveal melanoma will ultimately develop distant metastasis [3,4]. Unlike cutaneous melanoma, which metastasizes through lymphatic and hematogenous routes to multiple organs, including the lungs and lymph nodes, the eye lacks lymphatics and uveal melanoma develops and spreads by the hematogenous route and preferentially localizes in the liver. Metastatic disease of the liver remains the leading cause of death in patients with uveal melanoma and to date there are still no effective therapies for metastatic uveal melanoma. The phosphatidylinositol 3-OH kinase (PI3K)/AKT signaling pathway was found to be highly activated in uveal melanoma and the activation of AKT is associated with a higher risk of metastatic disease [3]. The Notch signaling pathway was also reported to be active in some but not all uveal melanoma cells, and blockage of Notch signaling reduced uveal melanoma growth and invasion [21]. The NF-κB signaling pathway has also been reported to be involved in various tumor metastases, such as breast adenocarcinoma, lung cancer and oral squamous cell carcinoma [27,30,31]. However, whether NF-κB signaling pathway is involved in uveal melanoma metastases still has not been well clarified. Using high concentrations FBS and the liver-produced cytokine, HGF, as chemoattractants, we found that blocking the NF-κB signaling pathway could inhibit the migration of uveal melanoma cells, suggested that NF-κB pathway might involve in uveal melanoma metastasis and targeting NF-κB pathway may be beneficial to patients with metastasis.

In addition, mouse studies suggested that NF-κB blockade by BAY 11-7072 also significantly induced apoptosis and inhibited tumor growth *in vivo*. Given the effects of NF-κB blockade decreasing cell migration, whether this also decreases uveal melanoma metastasis *in vivo* needs to be further tested in the future.

## 4. Experimental Section

### 4.1. Animals

BALB/c nude mice, male, four weeks of age, were purchased from Shanghai SLAC Laboratory Animal Co. Ltd (Shanghai, China). Mice were housed in the animal care facilities of the Ninth People’s Hospital, Shanghai Jiao Tong University School of Medicine under pathogen-free conditions. All experimental procedures were approved by the Laboratory Animal Care and Use Committees of the hospital.

### 4.2. Cell Lines and Reagents

The previously characterized human uveal melanoma cell lines OM431, VUP, SP6.5 and OCM1 were kindly provided by Professor John F. Marshall (Tumor Biology Laboratory, John Vane Science Centre, London, UK) [20,32]. ARPE-19 cells were obtained from the American Type Culture Collection (Manassas, VA, USA). All these established cells are composed of spindle and epithelioid cells, which were derived from primary tumors of choroidal and ciliary bodies. The specific NF-κB inhibitor BAY11-7082 (Calbiochem, San Diego, CA, USA) was reconstituted in dimethylsulphoxide (DMSO) (St. Louis, MO, USA) as a 100 mM stock solution and further diluted using PBS. Antibodies to IκBα, pIκBα, and caspase 3 were purchased from Cell Signaling Technology (Denvers, MA, USA). Antibodies to Bax, Bcl-2 and NF-κB p65 were obtained from BD Biosciences (San Diego, CA, USA). Anti-β-actin (clone AC-40) and DAPI were purchased from Sigma. IRDye 800CW goat anti-mouse secondary antibody and goat anti-rabbit secondary antibody were obtained from LI-COR Biotechnology (Lincoln, NE, USA).

### 4.3. Cell Culture and Cell Viability Assay

Uveal melanoma cells were cultured in Dulbecco’s modified Eagle medium (DMEM) (Invitrogen, Carlsbad, CA, USA) supplemented with penicillin (100 units/mL), streptomycin (100 μg/mL) and 10% (*v*/*v*) heat-inactivated fetal bovine serum (FBS) (Invitrogen). Cells were incubated at 37 °C in a humidified atmosphere containing 5% CO_2_. Uveal melanoma cell viability after BAY11-7082 treatment was determined with Cell Counting Kit-8 (Dojindo Molecular Technologies, Rockville, MD, USA) as described in our previous study [20]. Absorbance was measured by a microplate reader (VersaMax, Molecular Devices, Sunnyvale, CA, USA) at 450 nm and background absorbance measured at 630 nm was subtracted.

### 4.4. Apoptosis and Cell Cycle Analysis

To determine the apoptosis induced by BAY11-7082 stimulation, uveal melanoma cells (2 × 10^5^ cells/well) in 6-well plates were treated with 5 μM BAY11-7082 for 24 h and the same volume of DMSO (<0.05% *v*/*v*) was added into the culture medium of the control group. All uveal melanoma cells were collected, washed, and resuspended in 100 μL binding buffer and then incubated with 4 μL FITC-conjugated annexin-V (BD Pharmingen, San Diego, CA, USA) in the dark for 15 min at room temperature. All samples were immediately analyzed by flow cytometry, and the data were processed with FlowJo software. The cell cycle was analyzed by measuring the amount of propidium iodide (PI)-labeled DNA in ethanol-fixed cells. In brief, cells were treated for 24 h, harvested by trypsinization and fixed with cold 70% ethanol. Cells were then stained for total DNA content with PI/Rase staining buffer according to the manufacturer’s instructions. Cell cycle distribution was analyzed using a flow cytometer and ModFit software.

### 4.5. Western Blot Analysis

Related protein expression in uveal melanoma cells with BAY11-7082 treatment was determined by western blot. Protein extraction, quantitation and denaturation was performed as described previously [33]. After nonspecific sites in the PVDF membrane were blocked with a solution containing 5% non-fat milk powder in TBS/Tween20 (TBS/T) for 1 h at room temperature, the membrane was probed with antibodies against β-actin, IκBα, pIκBα, caspase 3, Bax and Bcl-2 in TBS/T containing 5% bovine serum albumin (BSA) overnight at 4 °C, and then incubated with diluted IRDye 800CW goat anti-mouse secondary antibody or goat anti-rabbit secondary antibody. Antibody-antigen complexes were then detected using the Odyssey^®^ Infrared Imaging system (LI-COR Biosciences, Lincoln, NE, USA).

### 4.6. Immunofluorescence Analysis

Tumor cells with or without 5 μM BAY11-7082 treatment were fixed with 4% paraformaldehyde and permeabilized with 0.1% Triton-X100 in PBS containing 1% BSA for 10 min. The cells were washed and blocked with 5% BSA for 1 h at room temperature. Cells were then incubated overnight at 4 °C with NF-κB p65 primary antibody (1:1000) (BD Biosciences), washed with PBS and incubated with Alexa Fluor^®^ 568 Goat Anti-Mouse IgG (H+L) (1:2000) (Invitrogen) for 1 h at room temperature. After washing, the cells were mounted with mounting medium containing DAPI (Sigma). Negative controls were run using the same protocol while replacing primary antibody with mouse IgG antibody. Stained cells were evaluated under a fluorescence microscope with a magnification of 200×.

### 4.7. Cell Clonogenic Assay

Cells were seeded into 6-well plates in triplicate at a density of 300 cells/well in 2 mL of medium containing 10% FBS. After 24 h, cultures were replaced with fresh medium containing 0.5% FBS (control) or the same medium containing 2.5 μM or 5 μM BAY11-7082 in a 37 °C humidified atmosphere containing 95% air and 5% CO_2_ and grown for three weeks. The cell clones were stained for 15 min with a solution containing 0.5% crystal violet and 25% methanol, followed by three rinses with tap water to remove excess dye. Colonies consisting of >50 cells were counted under a microscope with a magnification of 200×.

### 4.8. *In Vitro* Migration Assay

To measure cell migration *in vitro*, 8-μm pore size-culture inserts (Transwell, Corning, Lowell, MA, USA) were placed into 24-well culture plates, separating the upper and the lower chambers. The upper chamber contained cells in DMEM plus 1% FBS with or without 5 μM BAY11-7082, and the lower chamber contained DMEM plus 20% FBS or recombinant human hepatocyte growth factor (HGF) (20 ng/mL; R & D Systems, Minneapolis, MN, USA) in DMEM containing 1% FBS. Cells were incubated for 12 h at 37 °C in a humidified atmosphere containing 5% CO_2_. Non-migrated cells were removed from the upper surface of the transwell membrane with a cotton swab. Migrated cells remaining on the bottom surface were fixed and stained with a solution containing 0.5% crystal violet and 25% methanol, and then five independent visual fields under a light microscope were counted at a magnification of 25×. Results were expressed as the mean + SD. For each migration condition, three identical replicates were performed.

### 4.9. *In Vivo* Antitumor Activity

Animal protocols were approved by the Animal Care and Use Committee at Shanghai Jiao Tong University School of Medicine. For xenograft implantation, a total of 5 × 10^6^ OCM1 cells/mouse were injected subcutaneously into the back next to the right hind limb, and permitted to grow until palpable. Then mice were then randomly assigned into the control or treatment group and treatment was initiated. For the control group, 100 μL vehicle PBS containing the same volume of DMSO injection per mouse was injected subcutaneously peri-tumor every day for 14 days. For the treatment group, 1 μM BAY11-7082 in 100 μL PBS/mouse was injected in the same way as that in the control group. The day of cell inoculation was designated Day 0. Tumors were measured every 3 days with vernier calipers and tumor volumes were calculated according to the following formula:

(1) $$\text{tumor volume }({\text{mm}}^{3})=a\times b^{2}\times 0.52$$

where *a* is the longest diameter and *b* is the shortest diameter. Body weight of the mice was also recorded. At the end of the experiments, tumor-bearing mice were sacrificed, and tumors were weighed after being separated from the surrounding muscles and dermis. Finally, the tumors were fixed with 4% phosphate-buffered paraformaldehyde and embedded in paraffin.

### 4.10. TUNEL (Terminal Deoxynucleotidyl Transferase (TdT)-Mediated Nick End Labeling) Staining

For TUNEL staining, paraffin-embedded tumors from nude mice were assayed for DNA fragmentation according to the manufacturer’s instructions (Roche Molecular Biochemicals, Indianapolis, IN, USA) as described previously [33]. According to the manufacturer’s instructions of the *in situ* Cell Death Detection Kit supplied by Roche Applied Science, apoptotic cells were indicated by brown stains in the nucleus. Thus, we counted the apoptotic cells under light microscopy. Therefore, increased numbers of nucleus brown-stained cells suggested that more tumor cells underwent apoptosis. A total of 10 tissue sections were analyzed for each animal. All sections were examined by light microscopy and a total of 10 tissue sections were analyzed for each animal.

### 4.11. Statistical Analysis

A two-way repeated-measures analysis of variance (ANOVA) was used to test differences in tumor growth. One-way ANOVA was used to test differences in the mean growth inhibition, apoptosis rate and tumor weight. All results are expressed as mean ±/+ SD. All statistical tests were two-sided, and *p*-values less than 0.05 were considered statistically significant.

## 5. Conclusions

In summary, we have shown that NF-κB signaling is activated and promotes the malignant phenotype of uveal melanoma cells. Pharmacologic blockade of this pathway could inhibit uveal melanoma tumor growth and invasion. The *in vitro* and *in vivo* studies in our work suggest that therapies targeting the NF-κB pathway may be useful in new treatments for the uveal melanoma.
